# The Use of Time Flow Analysis to Describe Changes in Physical Ergonomic Work Behaviours Following a Cluster-Randomized Controlled Participatory Ergonomic Intervention

**DOI:** 10.1093/annweh/wxac058

**Published:** 2022-08-17

**Authors:** Charlotte Lund Rasmussen, Andreas Holtermann, Karel Hron, Dorothea Dumuid, Charlotte Diana Nørregaard Rasmussen

**Affiliations:** Department of Public Health and Nursing, Norwegian University of Science and Technology, Trondheim, Norway; Faculty of Physical Culture, Palacký University Olomouc, Olomouc, Czech Republic; National Research Centre for the Working Environment, Lersø Parkalle 105, 2100 Copenhagen, Denmark; National Research Centre for the Working Environment, Lersø Parkalle 105, 2100 Copenhagen, Denmark; Department of Mathematical Analysis and Applications of Mathematics, Palacký University Olomouc, Olomouc, Czech Republic; Alliance for Research in Exercise, Nutrition, and Activity (ARENA), Allied Health and Human Performance, University of South Australia, Adelaide, South Australia, Australia; National Research Centre for the Working Environment, Lersø Parkalle 105, 2100 Copenhagen, Denmark

**Keywords:** ergonomics, forward bending, kneeling, participatory ergonomic intervention, sedentary, time-use, time flow analysis, work behaviours

## Abstract

**Aim:**

Evaluations of participatory ergonomic interventions are often challenging as these types of interventions are tailored to the context and need of the workplace in which they are implemented. We aimed to describe how time flow analysis can be used to describe changes in work behaviours following a participatory ergonomic intervention.

**Method:**

This study was based on data from a two-arm cluster-randomized controlled trial with 29 childcare institutions and 116 workers (intervention: *n* = 60, control: *n* = 56). Physical behaviours at work were technically measured at baseline and 4-month follow-up. Physical behaviours were expressed in terms of relative work time spent forward bending of the back ≥30°, kneeling, active (i.e. walking, stair climbing and running) and sedentary. Average time flow from baseline to follow-up were calculated for both groups to investigate if work time was allocated differently at follow-up.

**Results:**

A total of 116 workers (60 in the intervention and 56 in the control group) had valid accelerometer at baseline and follow-up. The largest group difference in time flowing from baseline to follow-up was observed for forward bending of the back and time spent kneeling. Compared to the control, the intervention group had less time flowing from forward bending of the back to kneeling (intervention: +11 min day, control: +16 min day) and more time flowing from kneeling to sedentary behaviours (intervention: +15 min day, control: +10 min day).

**Conclusion:**

The results of this study showed that time flow analysis can be used to reveal changes in work time-use following a participatory ergonomic intervention. For example, the analysis revealed that the intervention group had replaced more work time spent kneeling with sedentary behaviours compared to the control group. This type of information on group differences in time reallocations would not have been possible to obtain by comparing group differences in work time-use following the intervention, supporting the usefulness of time flow analysis as a tool to evaluate complex, context-specific interventions.

**What’s Important About This Paper?**

Assessment of work behaviour changes following interventions are challenging, in part because group-level changes in behaviours may miss compensatory behavioural changes among individuals. This study demonstrates the use of time flow analysis to describe such trade-offs in the context of a participatory ergonomics intervention.

## Introduction

A participatory approach is recommended and commonly used for preventive workplace ergonomic interventions, particularly among occupational groups with high physical activity demands at work (Rivilis *et al.*, 2008). With this approach, workers take responsibility for identification, solution development and implementation of changes in the working environment to decrease risk factors in the work routines (van Eerd *et al.*, 2010). As a result, the workers’ ownership and involvement are enhanced, leading to locally targeted and tailored interventions to the needs, resources and context of the workers (Durlak and DuPre, 2008).

Despite the popularity of participatory interventions, their effectiveness has been questioned, particularly ergonomic interventions aimed to change physical ergonomic work behaviours (e.g. sitting, standing, and forward bending of the back) (Rivilis *et al.*, 2008; van Eerd *et al.*, 2010). This may be because participatory ergonomic interventions are specifically tailored to the various needs, context and physical demands of the workers and their work teams. Consequently, the aim, content and implementation of the intervention is often heterogeneous, which makes evaluation of its effectiveness challenging. For example, in a participatory ergonomic intervention comprising several work teams, one team might target and implement solutions related to reducing forward bending for the back, while another team focuses on reducing kneeling work. Subsequently, the intervention effect in terms of difference in average work-time spent on work behaviours between the two groups might be masked. Thus, evaluation of implementation and effectiveness of participatory ergonomic interventions ought to comprehend the different content and implementation across various work teams within an intervention group. A potential solution to this issue of evaluating participatory ergonomic interventions could be to use other analytical approaches for detecting behavioural changes, such as time flow analysis.

To the best our of knowledge, only one study has used time flow analysis for studying changes in time spent on different behaviours (Olds *et al.*, 2018). Using this technique, the authors where able to investigate how time flowed from one behaviour to another across the retirement transition. Similarly, time flow analysis can provide important insights required for evaluating participatory ergonomic interventions. In short, time flow analysis can reveal how much time was spent on work behaviours pre-intervention and post-intervention as well as the amount of time flowing between the work behaviours from pre- to-post-intervention. For example, using this technique allows assessment of what work time spent on kneeling is replaced with (e.g. sitting or forward bending of the back) and thus, not solely whether work time spent kneeling has been reduced. Considering that the health effects of reducing work time spent kneeling depends on whether the time is replaced with either sitting or forward bending of the back such information can provide essential insights to how an intervention was implemented. However, no study has used time flow analysis to describe work behaviour changes following a participatory ergonomic intervention.

We recently conducted a participatory ergonomic intervention at childcare institutions intended to reduce physical exertion at work and musculoskeletal pain by improving work tasks that the workers perceived as physically demanding (Rasmussen *et al.*, 2018). Thus, it is likely that the workers within each childcare institution chose different aims and implementation strategies to reduce physically demanding work. Accordingly, we consider this intervention to be a suitable “case” to investigate the utility of time flow analysis for describing changes in work behaviours following a participatory ergonomic intervention.

## Materials and methods

### Study design and participants

This study is based on data from a participatory ergonomic intervention in childcare institutions intended to reduce physical exertion and musculoskeletal pain among childcare workers (Rasmussen *et al.*, 2018). Full details on the study design, intervention development and data collection can be found elsewhere (Rasmussen *et al.*, 2018). In short, the study was cluster-randomized using a wait-list control and with childcare institutions forming the clusters. The childcare institutions were randomly assigned to two different arms (immediate/delayed intervention) whereby both groups received an intervention conducted over a 4-month period. The intervention began in the second half of 2017 and ended in July 2018 with the final data collection.

Eligibility criteria for the childcare institutions were: being an institution for children aged 0–3 years old; having a minimum of nine employees; and not having participated in an ergonomic course within the previous year. Within the Copenhagen Municipality, all childcare institutions that fulfilled these criteria were invited to participate. A total of 32 childcare institutions responded positively to the invitation; one was excluded for being too small and three were excluded as they recently had participated in an ergonomic course. Thus, a total of 29 institutions were eligible. All childcare workers from the randomized institutions were eligible for participation in the intervention, but participation in the evaluation of the trial was voluntary. Before entering the trial, all childcare workers were asked to sign informed consent. Additional details on the companies and workers involved have been reported previously (Rasmussen *et al.*, 2020).

### Randomization

The cluster randomization was balanced on institution size. Randomization was performed by an independent data manager using a computer-generated randomization in SAS (SAS Institute, Cary, NC, USA). Given the study design, blinding of participants was not possible. However, data collection was conducted by persons blinded to group allocation. Moreover, for this study, group allocation was not revealed to the authors until analyses and interpretations of the results were finalized, and thus we performed a blinded analysis.

### Ethics

The National Research Centre for the Working Environment has an institutional agreement with the Danish Data Protection Agency about procedures to treat confidential data (journal number 2015-41-4232), such as by securing data on a protected drive with limited access and making all individual data pseudonymous. The Danish National Committee on Biomedical Research Ethics (the local ethics committee of Frederiksberg and Copenhagen) has evaluated a description of the study and concluded that, according to Danish law as defined in Committee Act § 2 and § 1, the intervention described should not be further reported to the local ethics committee (reference number 16048606). The study is registered in the ISRCTN Registry (ISRCTN10928313).

### Data collection

This study was based on data collected at two time-points: baseline and 4-month follow-up. Electronic questionnaires were sent to all participants at both time-points, including measures of sociodemographic, smoking and general health (Ware, 1993). Anthropometric measures of body height, body weight and body mass index (body weight [kg]/(body height [m]^2^) were taken at baseline by trained clinical personnel. Technical measurements of physical and cardiovascular workloads were conducted for 3–5 days at both time-points. During the technical measurements, participants were asked to complete a diary reporting time at work, time in bed at night and non-wear time of the technical measurement devices.

### Intervention

Intervention activities were carried out by trained ergonomic consultants from the Work Environment Consultancy of Copenhagen (occupational therapists and physiotherapists). The intervention consisted of three participatory ergonomic workshops conducted over a 4-month period. The first workshop lasted for 3 h, where the workers identified work tasks which they considered to cause high physical workload and as risk factors for musculoskeletal pain. The end product of the first workshop was a prioritized list of three to four work tasks which the workers considered as physically demanding work tasks and an action plan for implementing solutions to reduce such work tasks. The two follow-up workshops lasted 1.5 h, each with the purpose of evaluating and potentially adjusting these solutions. More details about the intervention can be found elsewhere (Rasmussen *et al.*, 2018).

### Outcome measurement: work physical behaviours

Time spent in body postures (i.e. bending of the back), body position (i.e. standing, sitting, lying and kneeling) and activity types (i.e. walking, stair climbing and running) were assessed using data from four AX3 accelerometers (3-Axis Logging Accelerometer; Axivity Ltd., Newcastle Upon Tyne, UK). The accelerometers were fixed using double sided adhesive tape (3 M, Hair-Set, St. Paul, MN, USA) and transparent adhesive film (OPSITE FLEXIFIX; Smith & Nephew plc, Londong, UK) and placed on the upper back, right thigh, and right and left calf. Accelerometer data were downloaded using Actilife Software version 5.5 (Actigraph, Pensacola, FL, USA) and analysed using the custom-made MATLAB program Acti4 [The National Research Centre for the Working Environment, Denmark and The Federal Institute for Occupational Safety and Health, Germany (BAuA)] (Skotte *et al.*, 2014). The Acti4 program has been shown to separate body postures, positions and physical activity types with high sensitivity and specificity under semi-standardized (Korshøj *et al.*, 2014; Skotte *et al.*, 2014) and non-standardized (Stemland *et al.*, 2015) conditions.

### Study population

Daily work hours, leisure time and time in bed were defined from the participants’ self-reported diary information. Only workers with at least one day of valid accelerometer measurement of work periods at both baseline and follow-up were included in the analyses. A valid day was defined as having accelerometer measurements of ≥3.5 h or ≥75% of the individual’s average work time.

### Statistical analysis

Analyses were performed in R version 1.1.3 (Team, 2022), using the compositions (van den Boogaart and Tolosana-Delgado, 2008), robCompositions (Templ *et al.*, 2011), zCompositions (Palarea-Albaladejo and Martin-Fernandez, 2015) and lme4 (Bates *et al.*, 2015) packages.

Each childcare worker’s average daily work time was conceptualized as a five-part composition of: (i) standing while forward bending the trunk <30°; (ii) standing while forward bending the trunk ≥30°; (iii) kneeling; (iv) active (i.e. walking, stair climbing and running); and (v) sedentary (i.e. sitting and lying). Five workers had zero work time spent standing while forward bending the trunk or kneeling. These zero observations were assumed to be due to limited sampling and treated as rounded zeros (Martin-Fernandez *et al.*, 2012). Thus, they were imputed by expected values based on the information in the covariance structure of the observed dataset using the log-ratio Expectation-Maximization (EM) algorithm (Palarea-Albaladejo and Martín-Fernández, 2008).

Compositional means were calculated for the work composition to describe the central tendency of the data (Aitchison, 1982; Pawlowsky-Glahn *et al.*, 2015). These were obtained by computing the geometric mean of each part of the respective composition and then linearly adjusting them to sum up to the same total. In this case, we used both the workers’ average daily work time (i.e. 390 min) and 100 % as the total for all participants.

The work compositions at baseline and 4-month follow-up were expressed using isometric log-ratio (*ilr*) coordinates (Egozcue *et al.*, 2003) to enable statistical testing of intervention effect on the work composition. Specifically, the effects of the intervention on the work behaviour composition was evaluated using a multivariate mixed model, thereby taking the clustering of observations of workers within the same childcare institution (i.e., the 16 institutions) into account as well as the multivariate outcome for each individual (i.e. the set of *ilrs* expressing the composition at follow-up). As suggested by Bolker (2012), a stacked model was used to enable computation by the lme4 package which does not currently accept multivariate outcomes. The childcare institution was entered as a random effect. Intervention group was entered as fixed effect. Moreover, we adjusted for baseline composition by entering the ilrs as fixed effects. Conclusions about the effectiveness of the intervention were based on the effect of group and we set the statistical significance at *P* < 0.05 for a two-sided test.

### Time flow analysis

In short, time flow analytic is based on a succession of steps, defined by an initial state, a final state, and a quantity of change from initial to final states. Specifically in this study, this analysis was used for assessing how much time was spent in work behaviours pre-intervention (i.e. initial stage), post-intervention (i.e. final stage), and the amount of time flowing between behaviours from pre- to post-intervention (i.e. quantity). Please note that the time flow analysis is based on absolute values (min) and thus, is not based on relative information. Therefore, although the method still describes use of time, it is not a compositional data analysis.

Time flows were calculated for the following four physical behaviours: forward bending of the trunk >30°, kneeling, active (i.e. walking, stair climbing and running) and sedentary (i.e. sitting and lying). This was done for each childcare worker by comparing each 5-min interval at baseline with the corresponding 5-min interval at follow-up matched by day of the week. If multiple behaviours occurred within a 5-min interval, the behaviour taking up most time of the interval was defined as the “dominant” behaviour. A change in any physical behaviour during the 5-min interval was added to the time flow. For example, if at 10:00–10:05 AM on Monday at baseline the childcare worker was sedentary, but at 10:00–10:05 AM on Monday at follow-up the same worker was kneeling, 5 min was added to the time flow from sedentary to kneeling. Daily time flows were calculated for each worker and averaged across all workers in the intervention and control group, respectively. Finally, daily time flows were averaged across all workdays to express average weekly time flow for the two groups. For further information on how the time flow analysis was conducted, please contact the corresponding author, Charlotte Lund Rasmussen.

#### Example of interpreting results of the time flow analysis

**Figure 1.**
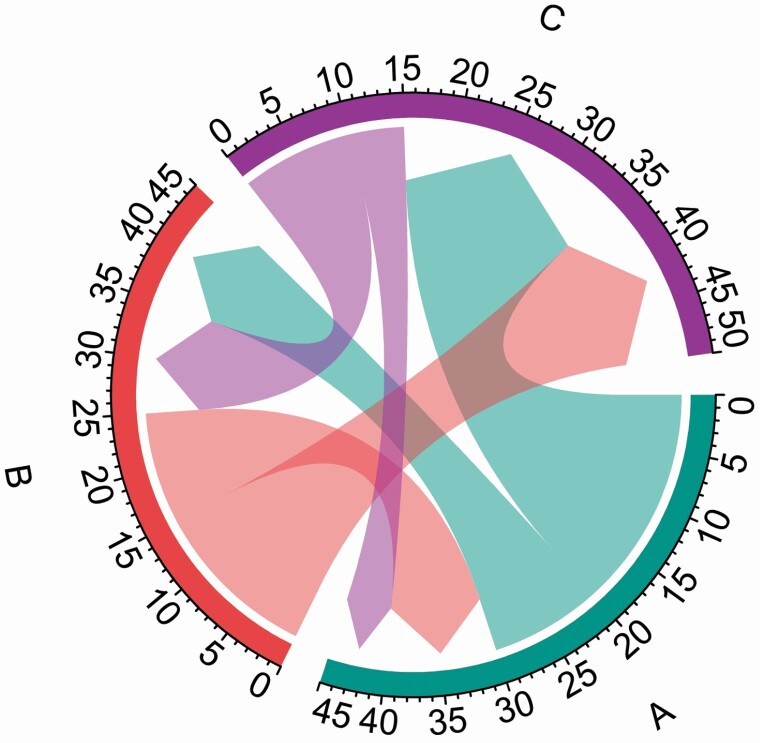
Example chord diagram of time flow from baseline to follow-up between behaviour A, B, and C.

Results of time flows were graphically presented in chord diagrams using the “circlize” package in R (Gu *et al.*, 2014). Chord diagrams are a specific type of flow diagrams, consisting of entities (nodes) that are connected by links (the flow). Importantly, the width of the link is proportional to the flow quantity. The chord diagram can be particularly useful for displaying inter-relationship in the flow, e.g. when time flows both from and to a behaviour. An example of a chord diagram is illustrated in Fig. 1, showing time flows between behaviour A, B and C from the initial state (i.e. baseline) to the final state (i.e. follow-up). The direction of the flow is indicated by the arrow (i.e. time flowing from baseline to follow-up) and the width of the arrow indicates how much time is flowing between the behaviours. Tick marks around the circle show the number of minutes flowing between behaviours. For example, focusing on behaviour A, we can see that 10 min and 20 min flowed from behaviour A to behaviour B and C, respectively. We can also see that 10 min and 5 min, flowed from behaviour B and C to behaviour A, respectively.

### Supplementary information

We further evaluated the intervention effect on relative work time spent in the different cardiorespiratory workloads. As we considered these investigations and results to be beyond the scope of the current paper, we chose to report these findings in Supplementary file 1 (available at *Annals of Work Exposures and Health* online).

## Results

**Figure 2.**
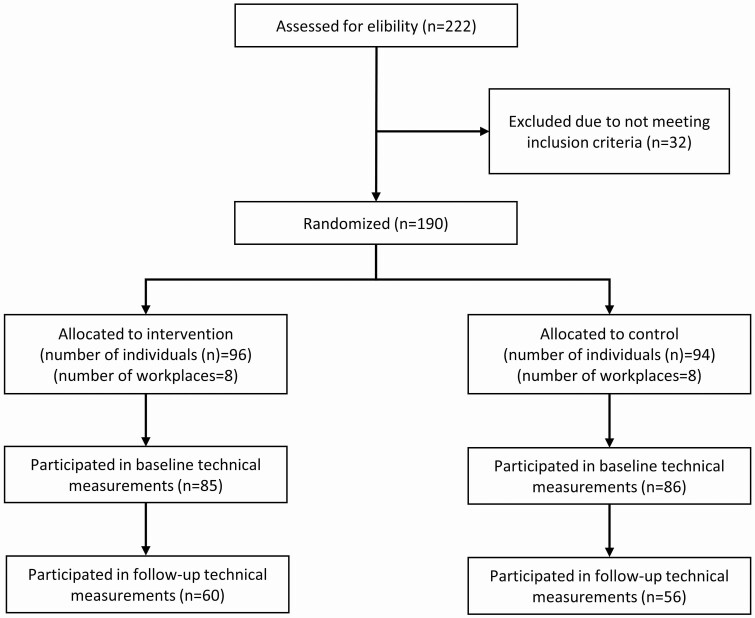
Flow of the study participants.

A total of 222 workers were assessed for eligibility, of which 190 were randomized to either intervention (*n* = 96) or control (*n* = 94) (Figure 2). A total of 85 and 86 participated in baseline measurements in the intervention and control group, respectively, of which 60 and 56 workers participated in follow-up measurements respectively. Thus, a total of 116 workers were included in the current study.

### Basic descriptive

The intervention and control group had similar demographic characteristics (Table 1). For both groups, most were female (85 % and 95 %, respectively) and non-smokers (72 % and 75 %, respectively). Mean age was 38.7 (SD = 12.0) and 40.3 (SD = 12.0) years and mean BMI was 26.0 (SD = 5.6) and 25.1 (SD = 5.8) for the intervention and control group, respectively. On average, the intervention group wore accelerometers for 3.7 days at baseline and for 3.6 days at follow-up. The control group wore accelerometers for 3.9 days at baseline and 3.6 days at follow-up.

**Table 1.** Characteristics of the study population (*n* = 116)

	Intervention *n* = 60	Control *n* = 56
Sex (female), *n* (%)	51 (85)	53 (95)
Age (years), mean (SD)	38.7 (12.0)	40.3(12.0)
BMI (kg cm^‐2^), mean (SD)	26.0 (5.6)	25.1 (5.8)
Non-smoker, *n* (%)	43 (72)	42 (75)
Overall health^A^, mean (SD)	2.1 (0.8)	2.0 (0.9)
Days with accelerometer measurements at baseline, mean (SD)	3.7 (1.0)	3.6 (1.0)
Days with accelerometer measurements at follow-up, mean (SD)	3.9 (0.8)	3.6 (1.0)

^A^Higher score indicates better self-reported health.

### Difference in compositional means

Overall, small differences were observed in compositional means between the groups at baseline and follow-up (Table 2). For both groups, the majority of work time was spent sedentary at baseline (177–186 min day). At follow-up, the intervention group had slightly less time kneeling (5 vs 8 min day) and more time active (60 vs 55 min day) compared to the control group. Results of the multivariate mixed model showed no significant intervention effect on the physical workload composition (*x*^2^ = 7.74, *P*-value = 0.17).

**Table 2.** Compositional means of physical workload composition at baseline and follow-up for the intervention and control group

	Baseline		Follow-up		Difference	
	Min. day	%	Min. day	%	Δ Min. day	Δ %
Intervention (*n* = 60)						
FB <30°	106	27	100	26	‐1	‐6
FB ≥30°	30	7	29	7	0	‐1
Kneeling	7	2	5	1	‐1	‐2
Active	61	16	60	15	‐1	‐1
Sedentary	186	48	196	50	2	10
Control (*n* = 56)						
FB <30˚	113	29	101	26	‐3	‐12
FB ≥30˚	24	9	25	9	0	1
Kneeling	6	1	8	2	1	2
Active	60	15	55	14	‐1	‐5
Sedentary	177	45	191	49	4	14

Sedentary behaviour defined as sitting and/or lying. Time-use was closed to the workers’ average daily work time (390 min) and 100 %.

FB, forward bending of the back; Active, walking, stair climbing and running.

### Time flow analysis

**Figure 3.**
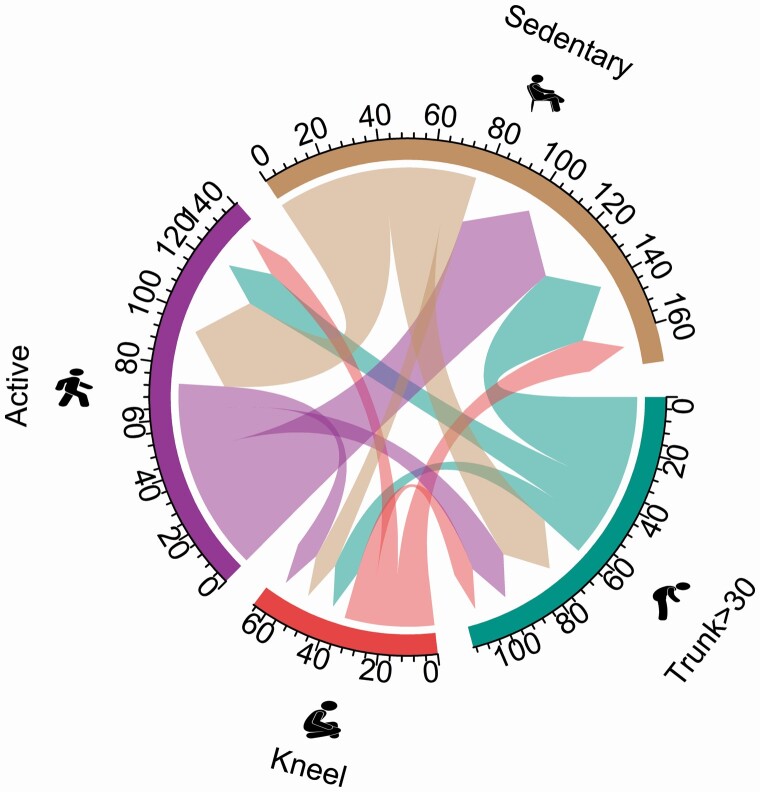
Chord diagram of time flow from baseline to follow-up between each work behaviour for the intervention group.

**Figure 4.**
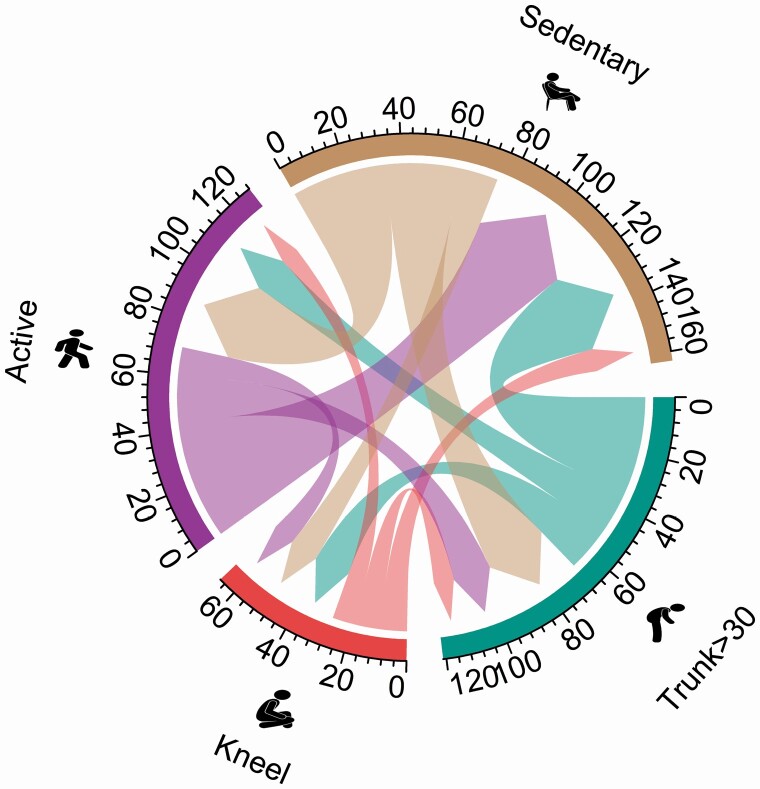
Chord diagram of time flow from baseline to follow-up between each work behaviour for the control group.

Table 3 and Figs. 3 and 4 shows the average time flow from baseline to follow-up for selected movement behaviours for the intervention and control group, respectively. Overall, the groups had similar average time flow from sedentary behaviour (i.e. intervention; ‐76 min day and control; ‐75 min day) and active time (intervention; ‐73 min day and control; ‐70 min day). For the intervention group, 63 min day flowed from time spent forward bending of the back >30° at baseline to sedentary behaviour (+35 min day), active time (+17 min day) and kneeling (+11 min day) at follow-up. In comparison, the control group had more time flowing from forward bending of the back (‐67 min day) at baseline to sedentary behaviour (+35 min day), active time (+16 min day) and kneeling (+16 min day) at follow-up. The intervention group had more work time flowing from kneeling (‐34 min day) compared to the control group (‐27 min day). For both groups, this time primarily flowed to sedentary behaviour (+15 min day and +10 min day) at follow-up. Difference in time flow for all behaviours between the two groups are shown in Table 3, revealing that the largest differences in time outflow was observed for forward bending of the back and kneeling time (‐4 min day and 7 min day, respectively).

**Table 3.** Time flow from baseline to follow-up between each movement behaviour for the intervention and control group and differences in time flow between the two groups in min day

Baseline (from)	Follow-up (to)				
	FB ≥30˚	Kneel	Active	Sedentary	Total outflow
Intervention (*n* = 60)					
FB ≥30°		11	17	35	63
Kneel	9		10	15	34
Active	16	10		47	73
Sedentary	25	10	41		76
Total inflow	50	31	68	97	
Control (*n* = 56)					
FB ≥30˚		16	16	35	67
Kneel	9		8	10	27
Active	17	10		43	70
Sedentary	28	13	34		75
Total inflow	54	39	58	88	
Difference in time flow between intervention and control					
FB ≥30°		‐5	1	0	‐4
Kneel	0		2	5	7
Active	‐1	0		4	3
Sedentary	‐3	‐3	7		1
Total inflow	‐4	‐8	10	10	

Sedentary behaviour defined as sitting and/or lying.

FB, forward bending of the back; Active,walking, stair climbing and running.

## Discussion

The aim of this study was to use a time flow analysis approach to describe changes in ergonomic work behaviours following a participatory ergonomic intervention among childcare workers. The results revealed that the largest group difference in work time flowing from baseline to follow-up was observed for forward bending of the back and time spent kneeling. Specifically, compared to the control group, the intervention group had less time flowing from forward bending of the back to kneeling, and more time flowing from kneeling to sedentary behaviour. These difference in changes in work behaviours between the groups were not evident when only comparing mean work-time spent in different behaviours at baseline and follow-up.

Our results suggest that time flow analysis can provide valuable insights to how time flows between various exposures from before to after a participatory ergonomic intervention. Such knowledge may be essential for understanding the effectiveness of complex participatory interventions. For example, it is not only valuable to know if an intervention reduces time in a harmful physical work behaviour (e.g. kneeling), but also if this reduction is replaced with more work time in another harmful behaviour (e.g. forward bending of the back) or to a non-harmful behaviour (e.g. sedentary behaviour). This is because the health effect of a reduction in a harmful work behaviour is likely to depend on whether it is replaced by just another harmful work behaviour or a non-harmful behaviour. Furthermore, this information provides valuable insight into the “black box” of participatory ergonomic interventions by going beyond assessment on an “average group level” to also gain knowledge about the mechanisms why the intervention was effective or not.

For these reasons, we think time flow analysis can provide useful knowledge for future implementation of participatory ergonomic interventions in childcare workers. That said, some limitations of time flow analysis should be noted. Time flow analysis cannot be used for traditional statistical testing of difference between groups and is at the current stage only suitable for a visual and explorative presentation of changes in time spent on various behaviours. Moreover, this method is not the most suitable approach to characterise variation in behaviours between workers or workplaces. If such analysis of variation is of interest, we suggest supplementing the time flow analysis with results obtained by mixed models. Finally, time flow analysis is based on comparing the exact same time interval at baseline/follow-up, e.g. Monday 9:00–9:05 AM before intervention to Monday 9:00–9:05 AM following intervention. Accordingly, an assumption when interpreting the results is that work tasks are performed at the same time and does not consider variation in work schedule. Thus, additional information on daily work schedules may be necessary to ensure that this assumption holds.

To the best of our knowledge, this is the first study to describe changes in ergonomic work behaviours following a participatory ergonomic intervention using time flow analysis. Although it is quite common that physical work demands (e.g. work time spent kneeling and forward bending of the back) are targeted by participatory ergonomic interventions, it is uncommon to evaluate changes in these work behaviours following the intervention (Rivilis *et al.*, 2008). Instead, the effectiveness of participatory ergonomic interventions is typically evaluated in terms of other outcomes, such as fatigue or musculoskeletal pain. We believe this could be caused by researchers acknowledging that traditional evaluation of pre-post average changes in work behaviours is not a suitable analytical approach to investigate effectiveness of complex participatory ergonomic interventions. Time flow analysis could be a method to close this research gap, enabling investigating of the exact changes in time-use before/after an intervention. For example, a participatory intervention may aim for work tasks that are typically performed while kneeling to be performed while sitting instead, which can be evaluated using our proposed method (i.e. revealing if kneeling time was replaced with sedentary time). Moreover, time flow analysis can be used to evaluate the effectiveness of implementation strategies to facilitate changes in behaviours. This insight could be gained by comparing time flow from baseline to post-intervention between workplaces/individuals using different implementation strategies.

### Strength and limitations

A major strength of our study was the use of accelerometry to measure work behaviours, and thus limit the risk of poor precision and misclassification associated with self-reported information. Moreover, the use of time flow analysis is a novel way to assess how the workers reallocated their work time in various work behaviours following a participatory ergonomic intervention. The blinded analysis of the study can also be considered a strength. A limitation of our study was the loss to follow-up (~60% in both groups) increasing the risk of selection bias. The use of 5-min intervals were based on the initial data-setup and has not been validated. On the one hand, the duration of the interval may have been too long to accurately capture the variation in behaviours that typically often occur in short bouts, e.g. kneeling and forward bending of the back. On the other hand, the interval duration may have been too short to capture work tasks that are not completed within 5 min, e.g. assisting children getting dressed. Consequently, we might have underestimated the time flowing between these behaviours. We encourage future studies to assess the potential influence of different interval durations on time flow analysis. Optimally, such studies would be combined with information on the average duration of each work task, to ensure that a suitable interval duration is chosen. Not all workers had measures of matching 5-min intervals of work periods at baseline and follow-up, and could therefore not be included in the time flow analysis. The lack of contextual information of the behaviours can be considered a limitation of our study as this is likely of importance to assess potential health effects. For example, although we were able to measure that a worker was sedentary, we are unable to state if this sedentary behaviour involved taking a rest break or sitting while taking care of children.

### Practical implications

When designing the evaluation of future participatory ergonomic interventions, we suggest to use time flow analysis as a supplementary method for understanding changes in work time spent on different behaviours following the intervention. This will allow prevention and intervention at workplaces to move beyond simply focussing on reducing a single exposure, to considering what ripple effects this reduction has on the remaining risk factors. For example, our time flow analysis revealed that the intervention seemed to result in work time spent kneeling being replaced with work time spent forward bending of the back (9 min day). As both kneeling and forward bending of the back are well-established ergonomic risk factors, the kneeling work time replaced with forward bending of the back is unlikely to prevent the workers for developing health issues. However, the time flow analysis also revealed that some kneeling work time was replaced with sedentary behaviour (15 min day), which suggests a reduction in physical workload and thus likely a beneficial time reallocation. For practice, this would imply that the workplace and workers should attempt to find work procedures or equipment which not only leads to reduction in work time spent kneeling, but also ensures that these work tasks are not being replaced by another risk factor (e.g. forward bending of the back).

We believe that the lessons from this study (and future studies) will enhance our understanding of the implementation and effect of participatory ergonomics interventions, which can be transferred to workers, workplaces, health and safety professionals, and researchers to improve future participatory ergonomic interventions among workers.

## Conclusion

The results of our study support the value of using time flow analysis for describing changes in ergonomic work behaviours following a participatory ergonomic intervention among childcare workers. When investigating average group-level differences following the intervention, no changes were found. However, when using time flow analysis, it became clear that the intervention group, compared to the control group, had less time flowing from one harmful work posture (forward bending of the back) to another (kneeling), and more time flowing from a harmful work posture (kneeling) to a non-harmful posture (sedentary behaviour). Such information can reveal if and to which extent participatory ergonomic interventions result in reduced time in harmful work behaviours and increased time in non-harmful work behaviours. This supports the usefulness of time flow analysis in describing trade-off in time-use following complex, context-specific participatory ergonomic interventions.

## Supplementary Material

wxac058_suppl_Supplementary_File

## Data Availability

The data underlying this article will be shared on reasonable request to co-authors Andreas Holtermann and Charlotte Diana Nørregaard Rasmussen.
