# Supplementary material for: The Use of Time Flow Analysis to Describe Changes in Physical Ergonomic Work Behaviours Following a Cluster-Randomized Controlled Participatory Ergonomic Intervention
Source: Ann Work Expo Health. 2022 Aug 17;66(9):1199–209. doi: 10.1093/annweh/wxac058 (PMC9664235; doi:10.1093/annweh/wxac058)
Supplement: wxac058_suppl_Supplementary_File [file wxac058_suppl_supplementary_file.docx]

**Supplementary file 1**

**­­­Title:** The use of time flow analysis to describe changes in physical ergonomic work behaviours following a cluster-randomized controlled participatory ergonomic intervention

**Authors:** Charlotte Lund Rasmussen^1,2,3^, Andreas Holtermann^3^, Karel Hron^4^, Dorothea Dumuid^5^, and Charlotte Diana Nørregaard Rasmussen^3^

^1^Department of Public Health and Nursing, Norwegian University of Science and Technology, Trondheim, Norway; ^2^Faculty of Physical Culture, Palacký University Olomouc, Czech Republic; ^3^National Research Centre for the Working Environment, Lersø Parkalle 105, 2100 Copenhagen, Denmark; ^4^Department of Mathematical Analysis and Applications of Mathematics, Palacký University Olomouc, Czech Republic; ^5^Alliance for Research in Exercise, Nutrition, and Activity (ARENA), Allied Health and Human Performance, University of South Australia, Adelaide, South Australia, Australia

**Method**

*Measurement of cardiorespiratory workload*

Heart rate was measured using Actiheart (Camntech, Cambridge, United Kingdom) placed at the chest at one of the two standardized positions (Brage et al., 2006), consisting of two electrodes connected by a short lead mounted to the skin by two standard electrocardiography pads (Blue sensor VL-00-S/25; Ambu A/S, Ballerup, Denmark) (Brage et al., 2005). Data were downloaded in the Actiheart software (Actiheart 4, version 4.0.116; CamNtech) and analysed using the program Acti4 to derive heart rate.

Heart rate data were filtered and checked for errors according to an earlier described protocol (Kristiansen et al., 2011). In brief, inter-beat intervals corresponding to <36 or >200 beats/min were considered as physiological outliers and excluded (Kristiansen et al., 2011). Moreover, heart rate measurements including > 50% beat error were excluded. Relative aerobic workload was estimated based on the heart rate reserve (%HRR); a well-established estimate of the aerobic workload on the body depending on the work demand and the individual’s cardiorespiratory fitness (Ilmarinen, 1992). Heart rate reserve was defined as the difference between estimated maximal heart rate (HR_max_) and sleeping heart rate (SHR) (HRR=HR_max_ – SHR) for each childcare worker (Karvonen et al., 1957). HR_max_ was determined by the Tanaka equation (Tanaka et al., 2001) and SHR was defined as the minimum heart rate of an average of ten beats/min during time in bed at night (Brage et al., 2004). The average relative aerobic workload was then calculated as the percentage of estimated HRR (average heart rate during work / HRR*100 = %HRR). Only workers with valid heart rate measurements at both baseline and follow-up were included in the evaluation of intervention effect on the cardiorespiratory workload composition (n=76).

*Statistical analysis*

Each childcare worker’s average daily work time spent in different cardiorespiratory workload were conceptualized as a 3-part work composition consisting of time spent at 1) HRR <20 %; 2) HRR 20-40 %; and 3) HRR ≥40 %. Compositional means were calculated for the work behaviour compositions to describe the central tendency of the data (Aitchison, 1986; Pawlowsky-Glahn et al., 2015).

The work composition at baseline and 4 month follow-up were expressed using isometric log-ratio (*ilr*) coordinates (Egozcue et al., 2003). This way, the 3-part cardiorespiratory workload composition was expressed as a set of 2 ilrs. The effect of the intervention on the cardiorespiratory workload composition was evaluated using a multivariate mixed model as described in the manuscript. That is, the childcare institution was entered as random effect. The log-transformed baseline composition and intervention group were entered as fixed effects. Conclusions about the effectiveness of the intervention was based on the group effect and we set the statistical significance at p<0.05 for a 2-sided test.

**Results**

*Compositional means and intervention effect*

The compositional mean of the cardiorespiratory workload composition showed that both groups spent most of their work time in HRR under 40 % both at baseline and follow-up (table S1). No intervention effect was observed for the cardiorespiratory workload composition (x^2^=0.95, p-value=0.62).

**Table S1.** Compositional mean of cardiorespiratory workload composition at baseline and follow-up for the intervention and control group.

|  | **Intervention (N=41)** | | | |
| --- | --- | --- | --- | --- |
|  | **Baseline** | | **Follow-up** | |
|  | *Min./day* | *%* | *Min./day* | *%* |
| HRR <20 % | 87 | 22 | 116 | 30 |
| HRR 20-40 % | 267 | 68 | 213 | 54 |
| HRR ≥40 % | 36 | 9 | 62 | 16 |
|  | **Control (N=35)** | | | |
|  | **Baseline** | | **Follow-up** | |
|  | *Min./day* | *%* | *Min./day* | *%* |
| HRR <20 % | 80 | 21 | 100 | 26 |
| HRR 20-40 % | 268 | 69 | 221 | 57 |
| HRR ≥40 % | 41 | 11 | 69 | 18 |

*HRR= heart rate reserve. Time-use was closed to the workers’ average daily work time (390 minutes) and 100 %.*
